# Approaches to α-amino acids via rearrangement to electron-deficient nitrogen: Beckmann and Hofmann rearrangements of appropriate carboxyl-protected substrates

**DOI:** 10.3762/bjoc.8.161

**Published:** 2012-08-29

**Authors:** Sosale Chandrasekhar, V Mohana Rao

**Affiliations:** 1 Department of Organic Chemistry, Indian Institute of Science, Bangalore 560 012, India

**Keywords:** amino acid, Beckmann rearrangement, Hofmann rearrangement, orthoacetate, trioxaadamantane

## Abstract

The titled approaches were effected with various 2-substituted benzoylacetic acid oximes **3** (Beckmann) and 2-substituted malonamic acids **9** (Hofmann), their carboxyl groups being masked as a 2,4,10-trioxaadamantane unit (an orthoacetate). The oxime mesylates have been rearranged with basic Al_2_O_3_ in refluxing CHCl_3_, and the malonamic acids with phenyliodoso acetate and KOH/MeOH. Both routes are characterized by excellent overall yields. Structure confirmation of final products was conducted with X-ray diffraction in selected cases. The final *N*-benzoyl and *N*-(methoxycarbonyl) products are α-amino acids with both carboxyl and amino protection; hence, they are of great interest in peptide synthesis.

## Introduction

The synthesis of α-amino acids remains of continuing interest for at least two reasons: Firstly, obtaining a particular amino acid via protein hydrolysis implies its separation from other amino acids (and their possible wastage, particularly on large scales). Secondly, the burgeoning of peptide science and protein engineering places significant demands on the supply of non-natural amino acids, which would generally be accessible only via synthesis. "Non-natural amino acids" also include the stereochemical alternative D-forms. This leads to additional challenges regarding chiral synthesis.

The challenges in the synthesis of α-amino acids essentially stem from the fact that the methods which are normally employed for the independent synthesis of the carboxyl and amine functionalities, are often mutually incompatible. Thus, for example, the oxidation of alcohols to carboxylic acids, or the Gabriel phthalimide synthesis cannot be performed in a routine way in the presence of the second functionality. Classical approaches essentially circumvented these limitations and ingenious improvements have evolved over the years [1].

Classical approaches continue to be investigated (most notably the Strecker reaction) [2], but recent developments focus on cycloadditions [3], rare amino acids [4], chiral catalysis [5], etc. It is noteworthy, however, that two of the most widely applied methods for the introduction of the amine group – the Beckmann [6–9] and the Hofmann [10–13] rearrangements – are not represented in these approaches. This is perhaps surprising, but understandable when the inherent instability of the requisite substrates is considered.

Thus, in the Beckmann approach, the oximation of β-keto acid derivatives would be complicated by competing deacylation, hydroxamic acid formation, etc. We are only aware of two reports [8–9] of the Beckmann rearrangement of β-keto ester oximes. A particular problem was the formation of isoxazolone byproducts, which apparently limited the synthesis to α,α-disubstituted derivatives. Likewise, the Hofmann approach would be complicated by the dubious stability of the malonamic acid substrates; a previous report indeed suggests that these suspicions are well-founded [13].

The key to success, therefore, is efficient carboxyl protection. Herein, we report a novel approach in which this is accomplished via the 2,4,10-trioxaadamantyl group, a proven masking group first described by Stetter [14] and Bohlmann [15] and extended by us [16]. The trioxaadamantyl group, a tricyclic orthoformate, may be introduced by trans-orthoesterification of an appropriate trimethyl orthoester with all-*cis* 1,3,5-trihydroxycyclohexane. This is accessible via Raney-Nickel catalyzed hydrogenation of phloroglucinol, the trans-orthoesterification being catalyzed by BF_3_ etherate.

The trioxaadamantyl group is extremely stable particularly under neutral and basic conditions [16]. Moreover, the trioxaadamantyl group can be selectively cleaved under acid catalysis, either to the carboxylic acid or directly to the ester, as demonstrated previously [16].

This carboxyl protection prevents the strategy from complications arising from the opportunistic side reactions observed in previous studies [8–9,13]. This has enabled the execution of two approaches to amino acids in which the key step is either the Beckmann or the Hofmann rearrangement, as described in the following.

## Results and Discussion

### Beckmann rearrangement approach

**Scheme 1:**
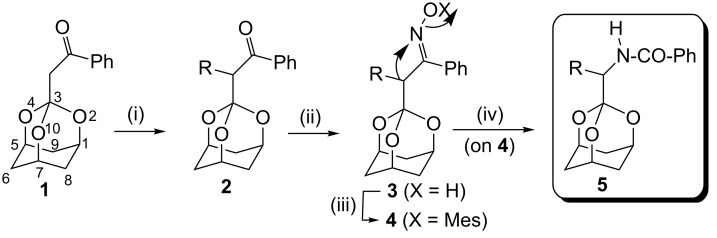
The transformation of the phenacyltrioxaadamantane **1** to the *N*-benzoyltrioxaadamantylmethylamine **5** via alkylation to **2** and the key Beckmann rearrangement of the oxime **3** to **5** ("Mes" represents methanesulfonyl; trioxaadamantane numbering is shown in **1**). Conditions: (i) NaH, THF, rt; RX, reflux; (ii) NH_2_OH·HCl, pyridine, EtOH, reflux; (iii) MeSO_2_Cl, Et_3_N, CH_2_Cl_2_, −10 °C; (iv) basic Al_2_O_3_, CHCl_3_, reflux.

The key substrate is the known 3-phenacyl-2,4,10-trioxaadamantane (**1**, Scheme 1) [14–16]. (An improved preparation of **1** is also presented herein). Ketone **1** was α*-*alkylated in high yields to various **2** via sodium hydride deprotonation and reaction of the resulting enolate with a variety of alkyl bromides or iodides. Alkyl derivatives **2** may be converted conventionally to the corresponding oximes **3**. The Beckmann rearrangement of **3** to the *N*-benzoyl derivative **5** is accomplished (generally) in high overall yields via a two-step sequence: Methanesulfonylation to compounds **4**, which are heated under reflux in CHCl_3_ in the presence of basic Al_2_O_3_ (Table 1) [17].

**Table 1:** Percentage yields of intermediate and final products of the transformation of **1** to **5** (Scheme 1) along with reaction times for their formation.

Entry	**2–5**	R–X	Yield (%) (reaction time/h)		

			**2**	**3**	**5**

1	**a**	MeI	96 (2)	77 (1)	98 (2)
2	**b**	*n*-PrI	89 (12)	82 (2)	94 (2)
3	**c**	*i*-BuI	79 (12)	74 (6)	93 (2)
4	**d**	PhCH_2_Br	97 (4)	84 (8)	96 (2)
5	**e**	CH_2_=CH–CH_2_Br	96 (4)	76 (3)	97 (2)
6	**f**	HC≡C–CH_2_Br	94 (4)	73 (11)	93 (2)

**Figure 1:**
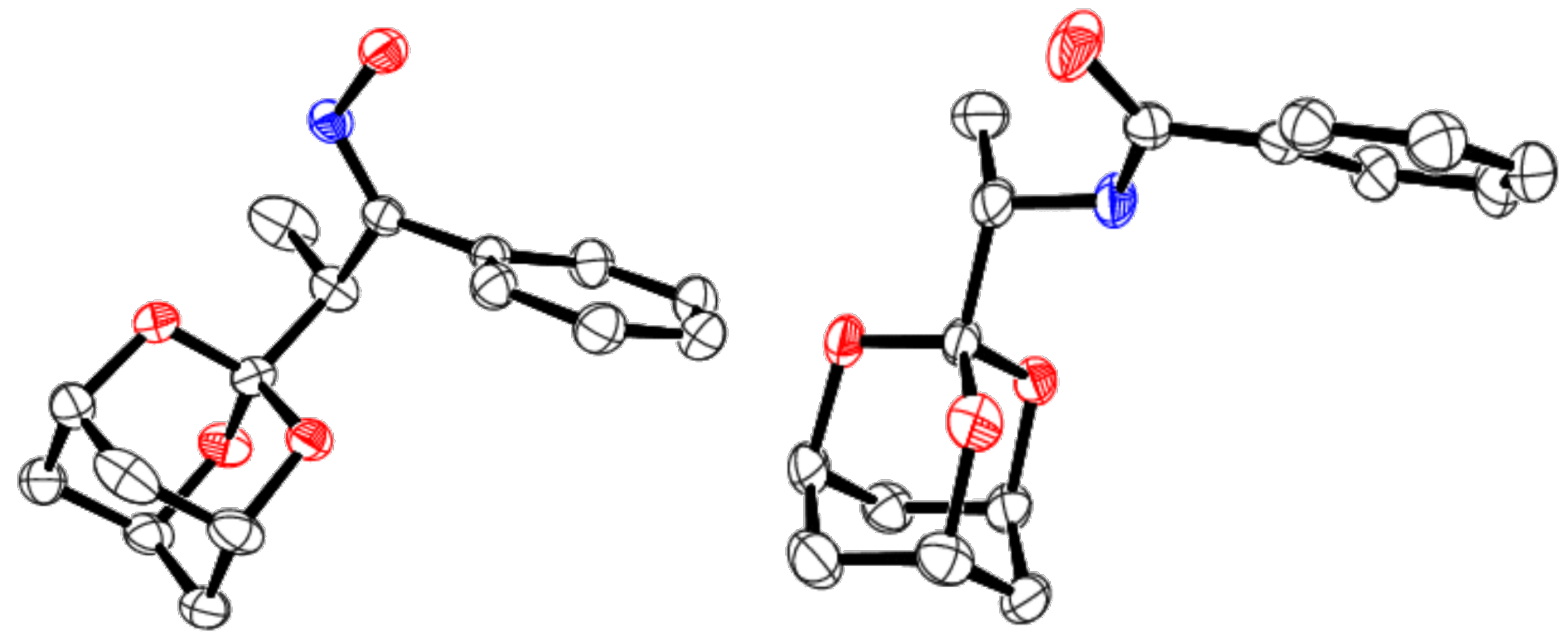
Crystallographic ORTEP diagram at 30% ellipsoidal probability of oxime **3a** (left) and amide **5a** (right, Scheme 1, Table 1) [18].

The formation of the desired *N*-benzoyl-trioxaadamantylmethylamines **5** indicates the *Z* geometry at the oximes **3** with the hydroxy and adamantylmethyl moieties being mutually *anti*. This apparently enables the Beckmann reaction – with its characteristic *anti*-migration – to occur smoothly. The presumed *Z* geometry has, in fact, been confirmed for the methyl derivative **3a** and the corresponding amide product **5a** by X-ray diffraction measurements (Figure 1 and Table 2) [18]. The observed oxime stereochemistry (*Z*) is possibly due to (putative) steric repulsion between the two bulk*y* α-substituents (R and trioxaadamantyl) and the hydroxy group in the alternative *E* isomer.

**Table 2:** Key crystallographic data for oxime **3a** and amide **5a**, representative substrate and product of the Beckmann rearrangement (Scheme 1, Figure 1) [18].

property	**3a**	**5a**

crystal system	orthorhombic	monoclinic
space group	*Pbca*	*P*1 2_1_/*n* 1
volume of unit cell (Å)^3^	2851.29 (17)	1431.9 (4)
*R*_all_, *R*_obs_	0.100, 0.048	0.079, 0.043
G.o.F.	0.929	1.021

The geometry around the partial double bond of the amide C–N linkage in **5a** is also *Z* (Figure 1). This is probably a consequence of steric repulsion between the *N*-substituent and the phenyl residue in the alternative *E* isomer. NMR spectra likewise did not indicate isomerism around the amide C–N bond in solution in any of the cases studied.

Attempted Beckmann rearrangement of **3** under several other conditions was largely unsuccessful. These conditions were: PCl_5_/dioxane, AlCl_3_/CHCl_3_, BF_3_·Et_2_O/CHCl_3_, *p*-toluenesulfonic acid/MeCN (all at reflux). The products of the transformations (**2–5**) described above were fully characterized spectroscopically (including high resolution mass spectrometry, HRMS) apart from X-ray diffraction in the cases mentioned earlier.

The final *N*-benzoyltrioxaadamantylmethylamine products **5** represent α-amino acids which are protected at both the carboxyl and the amine centers. The 2,4,10-trioxaadamantyl group may be cleaved with acid as reported previously [16]; alternatively, selective cleavage of the *N*-benzoyl group in base would furnish carboxyl-protected amino acids with a free amine function: all attractive tactical elaborations vis-à-vis peptide synthesis. Moreover, the transformation of **1** to **5** indicates that **1** functions as a glycine enolate equivalent with the benzoylamino group in **5** being introduced in umpolung fashion (reaction involves the migration to an electrophilic nitrogen center).

### Hofmann rearrangement approach

**Scheme 2:**
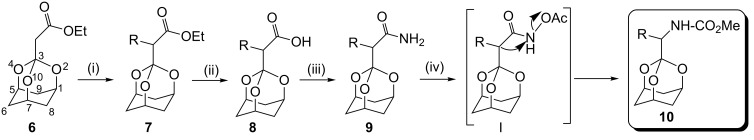
The transformation of the ethyl (trioxaadamantyl)acetate **6** to the *N*-(methoxycarbonyl)trioxaadamantylmethylamine **10**, via alkylation to **7**, hydrolysis to **8**, conversion to amide **9**, and its Hofmann rearrangement via putative *N*-acetoxy derivative **I**. Conditions: (i) (Me_3_Si)_2_N^−^Li^+^, THF, −78 °C, RX, 0.5 h; warm to rt, 6–8 h; (ii) *t*-BuO^−^K^+^, H_2_O, Et_2_O, 12–18 h; (iii) DCC, C_6_F_5_OH, EtOAc, 10 °C, 3 h, filter; filtrate: −20 °C, NH_3_, 0.5 h; rt, 6 h; (iv) PhI(OAc)_2_, KOH, MeOH, 5–10 °C, 15 min; then rt, 1 h.

Our strategy was based on the known ethyl trioxaadamantylacetate **6** (Scheme 2) [15]. Alkylation of this compound was accomplished via initial deprotonation with lithium hexamethyldisilazide in THF at −78 °C [19]. The resulting enolate was reacted with various alkyl bromides or iodides, initially at −78 °C and then at room temperature over 6–8 h. The α-alkyl derivatives **7** were formed in excellent yields (Table 3).

**Table 3:** Percentage yields of intermediate and final products of the transformation of **6** to **10** (Scheme 2) along with reaction times for their formation.^a^

Entry	**7**–**10**	R–X	Yield (%) (reaction time/h)			

			**7**	**8**	**9**	**10**

1	**a**	MeI	93 (6)	89 (18)	91	94
2	**b**	*n*-PrI	90 (8)	86 (18)	93	94
3	**c**	*i*-BuI	86 (8)	91 (18)	92	93
4	**d**	PhCH_2_Br	92 (6)	92 (18)	94	96
5	**e**	CH_2_=CH–CH_2_Br	94 (6)	88 (18)	90	91
6	**f**	4-(MeO)–C_6_H_4_CH_2_Br	91 (6)	90 (12)	92	95

^a^Isolated yields after chromatographic purification, except in the case of **8**.

The α-alkyl derivatives **7** were hydrolyzed with potassium *tert*-butoxide and water in ether [20], the corresponding acids **8** being obtained in excellent yields (Table 3). Conventional hydrolysis with aqueous KOH was unsuccessful, probably for steric reasons.

The conversion of acids **8** to their amides was carried out via initial activation with DCC (dicyclohexylcarbodiimide) and pentafluorophenol in ethyl acetate solution; this was followed by reaction with ammonia gas at −20 °C. The carboxamides **9**, substrates for the key Hofmann rearrangement step, were thus formed in excellent yields (Table 3).

The Hofmann rearrangement of carboxamides **9** was accomplished with one molar equivalent of phenyliodoso acetate [PhI(OAc)_2_] at 5–10 °C in methanolic KOH. The choice of the hypervalent iodine reagent was largely dictated by precedence [21–23]. The corresponding methyl carbamates **10** were obtained in excellent yields (Table 3).

The reaction may well occur via the putative *N*-acetoxy derivative **I** (up to now this has not been proven). The in situ trapping of the expected isocyanate intermediate (not shown) by methanol would explain the final formation of **10**.

**Figure 2:**
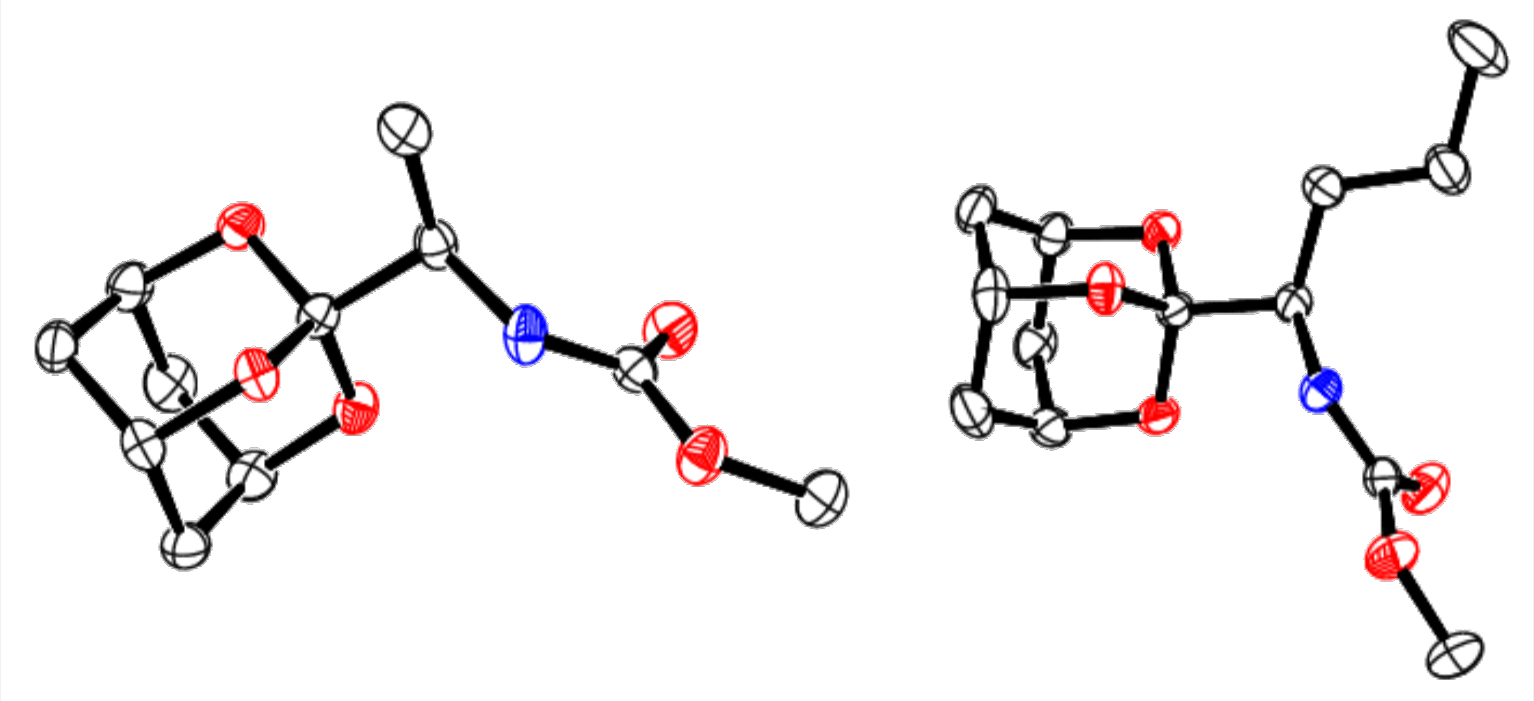
Crystallographic ORTEP diagrams at 30% ellipsoidal probability of carbamates **10a** (left) and **10b** (right, Scheme 2, Table 3) [24].

All products were generally purified chromatographically and fully characterized spectroscopically (including HRMS). In addition, two of the final carbamates (**10a** and **10b**) were confirmed by X-ray diffraction analysis (Figure 2 and Table 4) [24]. The carbamates **10** apparently evidenced restricted rotation around the amide C–N moiety (partial double-bond character) in the NMR as seen by twin peaks in certain cases [25].

**Table 4:** Key crystallographic data for carbamates **10a** and **10b**, which are representative products of the Hofmann rearrangement of amides **9** (Scheme 2, Figure 2) [24].

property	**10a**	**10b**

crystal system	orthorhombic	monoclinic
space group	*Pbca*	*P*1 2_1_/*n* 1
volume of unit cell (Å)^3^	2367.22 (5)	1397.00 (1)
*R*_all_, *R*_obs_	0.045, 0.034	0.048, 0.040
G.o.F.	1.064	1.092

In carbamates **10**, the substitution pattern α to the C=O group in **7–9** has shifted to the α-position next to the amino center. Therefore, in this strategy (Scheme 2) the enolate alkylation methodology has been co-opted to generate substitution α to the amino group, i.e., the (trioxaadamantyl)acetate **6** functions potentially as a glycine enolate equivalent.

It is also noteworthy that, as the Hofmann rearrangement involves migration to electron-deficient nitrogen [10–12], the amino group is being introduced in umpolung fashion in the strategy described above. Generally, the amino group is introduced nucleophilically, e.g., in the Gabriel method.

The trioxaadamantylmethylamine carbamates **10** also represent α*-*amino acids with both carboxyl and amino group protection. The methyl carbamates are clearly less robust than the BOC analogues which are frequently employed in peptide synthesis. However, the former do offer a measure of protection that can be selectively relinquished in base. The trioxaadamantyl group would stay unaffected under these conditions, but may be cleaved off with acid (possibly selectively). These conversions would be of considerable value in peptide synthesis strategy.

## Conclusion

Rationally designed, controlled approaches to α-amino acid synthesis via two classical reactions have been demonstrated. The key step is based on the Beckmann and the Hofmann rearrangements, two of the standard methods for the introduction of the amino functionality into molecules. The strategy is enabled by efficient carboxyl protection via the 2,4,10-trioxaadamantane moiety, which stabilizes the respective precursor substrates to opportunistic side reactions. Overall yields are excellent and the starting materials are accessible by standard methods. The α-alkyl groups are introduced via an enolate alkylation strategy, thus providing variety and the potential for molecular diversity. The key rearrangement steps are generally effected under fairly mild conditions.

## Experimental

**General comments.** Instruments employed: JASCO 410 (FTIR); Bruker AV-400 (NMR); Micromass Q-TOF AMPS MAX 10/6A (HRMS); Stuart SMP10 (melting point); Büchi Rotavapor R-200 (rotary evaporator).

The phenacyl **1** and acetate **6** derivatives of 2,4,10-trioxaadamantane were prepared as reported previously [14–16]. However, an improved oxidation procedure for the preparation of **1** is reported below. Upon aqueous work-up and extraction, extracts were generally dried with MgSO_4_ prior to evaporation of the solvent in vacuo in a rotary evaporator. All spectral details of compounds **2–5** and **7–10** have been collected in Supporting Information File 1.

**Improved preparation of phenacyltrioxaadamantane 1.** (2,4,10-Trioxaadamantylmethyl) phenyl carbinol [16] (not shown) was oxidized by 2-iodoxybenzoic acid as follows. The carbinol (0.500 g, 1.91 mmol) in EtOAc (10 mL) was treated with 2-iodoxybenzoic acid (1.070 g, 3.82 mmol), and the mixture was heated under reflux for 4 h. Then, it was cooled to 25 °C and stirred with solid NaHCO_3_ for 0.5 h. The insolubles were filtered off and the filtrate was concentrated in vacuo*.* The resulting residue was purified by column chromatography on grade 1 neutral alumina to obtain phenacyl derivative **1** (0.468 g, 1.80 mmol, 94%), identified by mp (105–106 °C) and spectral comparison [16].

**Alkylation of 1 to 2.** NaH (1.2 mmol) was washed with Na/dry hexane, covered with dry THF (1.0 mL) and treated with a solution of **1** (1.0 mmol) in dry THF (6.0 mL) at 0 °C. The mixture was stirred for 10 min, treated with the alkyl halide (RX, 1.2 mmol) and heated under reflux for the indicated time (Table 1). The reaction mixture was cooled to room temperature, worked-up with ice-water and extracted with EtOAc. Purification by column chromatography on grade 1 neutral alumina furnished **2**.

**Oximation of 2 to 3.** Alkylketones **2** were converted to the corresponding oximes **3** with NH_2_OH·HCl-pyridine by standard procedures [26] and purified by crystallization (solids).

**Oxime methanesulfonates 4.** Oximes **3** (1.0 mmol) in CH_2_Cl_2_ (3.0 mL) were cooled to −10 °C and treated with Et_3_N (1.2 mmol) followed by MeSO_2_Cl (1.5 mmol). The mixtures were stirred for 2 h and washed with water, etc. The crude mixtures were purified by column chromatography on grade 1 neutral alumina to obtain **4**.

**Beckmann rearrangement of 4 to final amides 5** [17]. Mesylates **4** (1.0 mmol) in CHCl_3_ (5.0 mL) were treated with basic alumina (0.25 g) and the mixtures were heated under reflux for 2 h. After cooling to room temperature, the alumina was filtered off and the solvent was removed in vacuo*.* The residues were purified by column chromatography on grade 1 neutral alumina to obtain the pure amides **5**.

**Alkylation of ethyl trioxaadamantylacetate 6 to 7** [19]. A stirred solution of **6** (2.0 mmol) in dry THF (8.0 mL) at −78 °C was treated with lithium hexamethyldisilazide (1.0 M in THF, 2.4 mL, 2.4 mmol). After 0.5 h, the resulting enolate solution was treated with the alkyl halide (2.2 mmol) over ~2 min and stirred for 0.5 h at −78 °C. The mixture was warmed to room temperature, stirred as indicated (Table 3), worked up with aq NH_4_Cl and extracted with EtOAc etc., followed by SiO_2_ column chromatography.

**Hydrolysis of ester 7 to 8** [20]. A stirred mixture of *t*-BuO^−^K^+^ (8.0 mmol) in Et_2_O (16.0 ml) at 0 °C was treated with water (2.0 mmol). After 5 min, the slurry was treated with ester **7** (1.0 mmol) and the mixture stirred at room temperature as indicated (Table 3). The mixture was treated with iced water (~5 mL), and the aqueous layer separated and acidified (conc. HCl). Extraction with EtOAc, etc., furnished acids **8**, used as such for the next step.

**Conversion of the carboxylic acid 8 to the amide 9.** Acid **8** (0.5 mmol) in EtOAc (6.0 mL) was treated with C_6_F_5_OH (0.55 mmol) followed by DCC (0.6 mmol), all at 10 °C with stirring. After 2 h, the mixture was filtered to remove dicyclohexylurea. The filtrate was cooled to −20 °C and NH_3_ gas bubbled in over 0.5 h. The mixture was stirred at room temperature for 6 h and the volatiles removed in vacuo. The residue was treated with water and worked up with CH_2_Cl_2_, etc., the crude products being purified by neutral Al_2_O_3_ (grade 1) column chromatography.

**Hofmann rearrangement of 9 to final carbamates 10** [21–23]. A stirred solution of **9** (0.2 mmol) in MeOH (5.0 mL) at 5–10 °C was treated with PhI(OAc)_2_ (0.2 mmol, one batch). After 15 min, the mixture was warmed to room temperature, and stirred for 1 h. The volatiles were removed in vacuo and the residue was treated with water and worked up with CH_2_Cl_2_, etc. The resulting crude **10** were purified by SiO_2_ column chromatography.

## Supporting Information

File 1 Characterization data.
