# Supplementary material for: Approaches to α-amino acids via rearrangement to electron-deficient nitrogen: Beckmann and Hofmann rearrangements of appropriate carboxyl-protected substrates
Source: Beilstein J Org Chem. 2012 Aug 29;8:1393–9. doi: 10.3762/bjoc.8.161 (PMC3458766; doi:10.3762/bjoc.8.161)
Supplement: File 1 — Characterization data. [file Beilstein_J_Org_Chem-08-1393-s001.pdf]

Supporting Information

for

Approaches to α-amino acids via rearrangement to electron-deficient nitrogen: Beckmann and Hofmann rearrangements of appropriate carboxyl-protected substrates

Sosale Chandrasekhar* and V. Mohana Rao

Address: Department of Organic Chemistry, Indian Institute of Science, Bangalore 560 012, India

Email: Sosale Chandrasekhar - sosale@orgchem.iisc.ernet.in

*Corresponding author

Characterization data

Melting points (Mp) are uncorrected. IR values are for neat samples and are quoted in cm^{-1}. NMR spectra were recorded in $CDCl_3$ solution with tetramethylsilane as internal standard at either 400 MHz (δ_H) or 100 MHz (δ_C); the coupling constants (J) are in Hz. Abbreviations: s (singlet); d (doublet); t (triplet); q (quartet); br (broad); dd (doublet of doublet), etc.

α-Alkyl-α-(2,4,10-trioxaadamant-3-yl)methyl phenyl ketones **2**

Methyl derivative **2a**. Mp 110–112 °C; υ_{max} 2956, 1686, 1133; δ_H 8.03–8.01 (2H, d, J 7.5), 7.53–7.50 (1H, m), 7.44–7.41 (2H, t, J 7.5), 4.36 (3H, br s), 3.88–3.83 (1H, q, J 7.0), 2.57–2.54 (3H, d, J 12.5), 1.66–1.63 (3H, d, J 12.5), 1.28–1.27 (3H, d, J 7.0); δ_C 199.24, 138.29,

132.35, 129.00, 128.02, 110.26, 68.19, 47.90, 32.77, 11.27; HRMS: 297.0996 (calcd. for $C_{16}H_{18}O_4$ + Na 297.1103).

n-Propyl derivative **2b**. Mp 120–122 °C; υ_{max} 2957, 1686, 1133; δ_H 8.05–8.02 (2H, m), 7.55–7.31 (3H, m), 4.37 (3H, br s), 3.81–3.76 (1H, d d, *J* 3.3, 11.1), 2.58–2.54 (3H, d, *J* 12.9), 2.09–1.96 (1H, m), 1.86–1.73 (1H, m), 1.67–1.63 (3H, d, *J* 12.9), 1.25–1.10 (2H, m), 0.84 (3H, t, *J* 7.2); δ_C 199.38, 139.39, 132.35, 128.85, 128.13, 110.34, 68.24, 53.80, 32.82, 29.03, 21.05, 14.20; HRMS: 325.1392 (calcd. for $C_{18}H_{22}O_4$ + Na 325.1416).

Isobutyl derivative **2c**. Mp 156–158 °C; υ_{max} 2957, 1686, 1132; δ_H 8.06–8.04 (2H, m), 7.55–7.41 (3H, m), 4.36 (3H, br s), 3.89–3.84 (1H, dd, *J* 3.3, 11.1), 2.58–2.54 (3H, d, *J* 12.9), 2.12–2.02 (1H, m), 1.74–1.55 (4H, m), 1.44–1.25 (1H, m), 0.83–0.79 (distinct diastereotopic Me's: 2 × 3H, 2 × d, *J* 4.8 Hz); δ_C 199.21, 139.26, 132.31, 128.89, 128.14, 110.48, 68.24, 51.81, 35.70, 32.82, 26.07, 23.66, 21.88; HRMS: 339.1768 (calcd. for $C_{19}H_{24}O_4$ +Na 339.1572).

Benzyl derivative **2d**. Mp 202–204 °C; υ_{max} 2958, 1686, 1314, 1131; δ_H 7.81–7.79 (2H, m), 7.44–7.01 (8H, m), 4.40 (3H, br s), 4.10–4.05 (1H, dd, *J* 3.0, 11.1), 3.39–3.16 (2H, m), 2.61–2.57 (3H, d, *J* 12.9), 1.69–1.65 (3H, d, *J* 12.9); δ_C 198.58, 139.78, 139.39, 132.13, 129.02, 128.61, 128.16, 127.93, 125.81, 110.13, 68.36, 55.82, 32.82, 32.66; HRMS: 373.1426 (calcd. for $C_{22}H_{22}O_4$ + Na 373.1416).

Allyl derivative **2e**. Mp 133–136 °C; υ_{max} 2958, 1686, 1132; δ_H 8.03–8.00 (2H, m), 7.54–7.31 (3H, m), 5.73–5.60 (1H, m), 5.01–4.83 (2H, m), 4.37 (3H, br s), 3.89–3.84 (1H, dd, *J* 3.6, 10.8), 2.85–2.54 (5H, m), 1.67–1.63 (3H, d, *J* 12.6); δ_C 198.28, 139.22, 135.87, 132.34, 128.88, 128.09, 116.30, 110.06, 68.29, 53.43, 32.79, 31.07; HRMS: 323.1234 (calcd. for $C_{18}H_{20}O_4$ + Na 323.1259).

Propargyl derivative **2f**. Mp 193–194 °C; υ_{max} 3285, 2961, 1686, 1315, 1130; δ_H 8.07–8.04 (2H, m), 7.56–7.42 (3H, m), 4.35 (3H, br s), 4.06–4.01 (1H, dd, *J* 3.3, 11.1), 3.02–2.92 (1H, m), 2.73–2.65 (1H, d t, *J* 3.0, 16.5), 2.56–2.51 (3H, d, *J* 13.5), 1.76 (1H, t, *J* 2.7), 1.66–1.62 (3H, d, *J* 13.5); δ_C 197.10, 138.88, 132.56, 129.06, 128.09, 109.43, 82.42, 68.77, 68.35, 52.96, 32.68, 16.11; HRMS: 321.1105 (calcd. for $C_{18}H_{18}O_4$ + Na 321.1103).

Ketoximes **3**

Methyl derivative **3a**. Mp 186–188 °C; υ_{max} 3257, 2951, 1592, 1315, 1133; δ_H 7.39–7.30 (5H, m), 4.32 (3H, br s), 3.04–2.97 (1H, q, *J* 7.2), 2.54–2.50 (3H, d, *J* 12.6), 1.67–1.62 (3H, d, *J* 12.6), 1.25–1.22 (3H, d, *J* 7.5); δ_C 158.41, 134.61, 128.14, 127.45, 127.20, 110.51, 67.92, 48.15, 32.70, 12.43; HRMS: 312.1485 (calcd. for $C_{16}H_{19}NO_4$ + Na 312.1212).

n-Propyl derivative **3b**. Oil; υ_{max} 3306, 2958, 1634, 1314, 1133; δ_H 7.43–7.27 (5H, m), 4.32 (3H, br s), 2.84–2.79 (1H, dd, *J* 4.2, 10.8), 2.56–2.52 (3H, d, *J* 12.3), 1.82–1.30 (7H, m), 0.89 (3H, t, *J* 7.5); δ_C 157.13, 134.85, 128.25, 127.80, 127.41, 110.75, 68.09, 54.00, 32.80, 29.42, 20.76, 14.03; HRMS: 340.1535 (calcd. for $C_{18}H_{23}NO_4$ +Na 340.1525).

Isobutyl derivative **3c**. Mp 160–161 °C; υ_{max} 3333, 2958, 1581, 1352, 1131; δ_H 7.38–7.30 (5H, m), 4.35 (3H, br s), 2.97–2.93 (1H, dd, *J* 3.2, 12.0), 2.57–2.54 (3H, d, *J* 12.8), 1.77–1.47 (6H, m), 0.95–0.87 (2x3H, d, *J* 6.4); δ_C 157.66, 134.56, 128.30, 127.91, 127.52, 110.85, 68.16, 51.89, 35.91, 32.83, 25.25, 23.96, 21.27; HRMS: 354.1683 (calcd. for $C_{19}H_{25}NO_4$ + Na 354.1681).

Benzyl derivative **3d**. Mp 189–191 °C; υ_{max} 3328, 2959, 1581, 1315, 1131; δ_H 7.26–6.96 (10H, m), 4.42 (3H, br s), 3.35–3.03 (3H, m), 2.64–2.60 (3H, d, *J* 12.6), 1.70–1.66 (3H, d, *J* 12.6); δ_C 155.93, 140.07, 135.53, 129.52, 128.16, 127.79, 127.76, 127.38, 125.86, 110.78, 68.28, 55.73, 33.94, 32.90; HRMS: 388.1522 (calcd. for $C_{22}H_{23}NO_4$ + Na 388.1525).

Allyl derivative **3e**. Mp 120–123 °C; υ_{max} 3311, 2957, 1574, 1314, 1132; δ_H 7.42–7.29 (5H, m), 5.92–5.78 (1H, m), 5.15–5.03 (2H, m), 4.37 (3H, br s), 2.97–2.92 (1H, dd, *J* 3.6, 11.4), 2.65–2.42 (5H, m), 1.67–1.63 (3H, d, *J* 12.9); δ_C 156.99, 136.49, 134.60, 128.23, 127.97, 127.51, 116.49, 110.54, 68.22, 53.84, 32.82, 31.52; HRMS: 338.1368 (calcd. for $C_{18}H_{21}NO_4$ + Na 338.1368).

Propargyl derivative **3f**. Mp 168–169 °C; υ_{max} 3410, 3292, 2960, 2930, 1576, 1312, 1127; δ_H 7.49–7.30 (5H, m), 4.35 (3H, br s), 3.13–3.07 (1H, dd, *J* 4.2, 12.0), 2.76–2.52 (5H, m), 2.02 (1H, t, *J* 2.7), 1.66–1.62 (3H, d, *J* 12.9); δ_C 156.48, 134.21, 128.17, 128.07, 127.57, 109.89, 82.72, 69.72, 68.24, 53.23, 32.72, 17.11; HRMS: 336.1203 (calcd. for $C_{18}H_{19}NO_4$ + Na 336.1212).

Ketoxime *O*-methanesulfonates (mesylates) **4**

Methyl derivative **4a**. Mp 122–124 °C; υ_{max} 2960, 1612, 1367, 1182; δ_H 7.38–7.35 (3H, m), 7.28–7.25 (2H, m), 4.32 (3H, br s), 3.17–3.13 (4H, m), 2.53–2.48 (3H, d, *J* 13.2), 1.66–1.62 (3H, d, *J* 13.2), 1.32–1.30 (3H, d, *J* 7.5); δ_C 168.82, 132.01, 128.83, 127.60, 127.58, 110.03, 68.27, 48.53, 36.47, 32.74, 12.12; HRMS: 390.1041 (calcd. for $C_{17}H_{21}NO_6S$ + Na 390.0987).

n-Propyl derivative **4b**. Mp 119–121 °C; υ_{max} 2960, 1612, 1367, 1182; δ_H 7.38–7.27 (5H, m), 4.32 (3H, br s), 3.14 (3H, s), 3.02–2.97 (1H, dd, *J* 4.2, 10.8), 2.53–2.49 (3H, d, *J* 13.2), 1.87–1.70 (2H, m), 1.66–1.62 (3H, d, *J* 13.2), 1.55–1.22 (2H, m), 0.96 (3H, t, *J* 7.5); δ_C 167.84, 132.08, 128.86, 127.63, 127.59, 110.11, 68.28, 54.24, 36.42, 32.73, 28.06, 20.59, 13.93; HRMS: 418.1308 (calcd. for $C_{19}H_{25}NO_6S$ + Na 418.1300).

Isobutyl derivative **4c**. Mp 127–128 °C; υ_{max} 2958, 2930, 1581, 1130; δ_H 7.39–7.35 (3H, m), 7.29–7.26 (2H, m), 4.31 (3H, br s), 3.13–3.09 (4H, m), 2.52–2.49 (3H, d, *J* 13.2), 1.81–1.56 (6H, m), 0.98–0.93 (2 × 3H, d, *J* 6.4); δ_C 167.95, 131.97, 128.84, 127.66, 127.60, 110.17,

68.27, 52.15, 36.38, 34.62, 32.70, 25.28, 23.75, 21.03; HRMS: 432.1464 (calcd. for $C_{20}H_{27}NO_6S$ + Na 432.1457).

Benzyl derivative **4d**. Mp 136–139 °C; υ_{max} 2963, 1624, 1364, 1130; δ_H 7.28–7.17 (8H, m), 7.02–6.98 (2H, m), 4.38 (3H, br s), 3.38–3.27 (2H, m), 3.13–3.05 (1H, dd, *J* 12.0, 14.1), 2.92 (3H, s), 2.57–2.53 (3H, d, *J* 12.6), 1.70–1.67 (3H, d, *J* 12.6); δ_C 165.58, 138.99, 132.91, 129.41, 128.86, 128.40, 127.56, 127.42, 126.27, 110.13, 68.41, 56.13, 36.21, 32.83, 32.79; HRMS: 466.1305 (calcd. for $C_{23}H_{25}NO_6S$ + Na 466.1300).

Allyl derivative **4e**. Oil; υ_{max} 2960, 1581, 1364, 1182, 1130; δ_H 7.37–7.27 (5H, m), 5.90–5.77 (1H, m), 5.19–5.08 (2H, m), 4.33 (3H, br s), 3.11–3.08 (4H, m), 2.71–2.48 (5H, m), 1.68–1.63 (3H, d, *J* 12.9); δ_C 166.64, 135.47, 132.18, 128.86, 127.55, 127.53, 117.11, 109.84, 68.30, 53.97, 36.37, 32.62, 30.50; HRMS: 416.1140 (calcd. for $C_{19}H_{23}NO_6S$ + Na 416.1144).

Propargyl derivative **4f**. Oil; υ_{max} 3289, 2959, 1614, 1315, 1130; δ_H 7.37 (5H, s), 4.31 (3H, br s), 3.28–3.24 (1H, dd, *J* 6.0, 11.4), 3.14 (3H, s), 2.81–2.63 (2H, m), 2.52–2.48 (3H, d, *J* 12.6), 2.05 (1H, t, *J* 2.7), 1.68–1.62 (3H, d, *J* 12.6); δ_C 165.50, 132.03, 128.99, 127.71, 127.51, 109.26, 81.91, 70.42, 68.38, 53.51, 36.50, 32.63, 16.48; HRMS: 414.0981 (calcd. for $C_{19}H_{21}NO_6S$ + Na 414.0987).

N-Benzoyl 1-(2,4,10-trioxaadamant-3-yl)methylamines **5** (Beckmann reaction products)

Methyl derivative **5a**. Mp 190–191 °C; υ_{max} 3444, 2957, 1652, 1133; δ_H 7.81–7.78 (2H, m), 7.51–7.40 (3H, m), 6.44–6.41 (1H, br d, *J* 8.1), 4.43 (3H, br s), 4.35–4.25 (1H, m), 2.62–2.58 (3H, d, *J* 12.6), 1.74–1.70 (3H, d, *J* 12.6), 1.27–1.25 (3H, d, *J* 6.6); δ_C 166.73, 135.05, 131.16, 128.38, 127.02, 109.94, 68.21, 51.11, 32.95, 14.76; HRMS: 312.1216 (calcd. for $C_{16}H_{19}NO_4$ + Na 312.1212).

n-Propyl derivative **5b**. Mp 196–198 °C; υ_{max} 3329, 2959, 1656, 1314, 1132; δ_H 7.83–7.80 (2H, m), 7.52–7.41 (3H, m), 6.25–6.22 (1H, br d, *J* 9.6), 4.41 (3H, br s), 4.34–4.26 (1H, m),

2.61–2.56 (3H, d, *J* 12.9), 1.91–1.78 (2H, m), 1.72–1.68 (3H, d, *J* 12.9), 1.51–1.30 (2H, m), 0.93 (3H, t, *J* 7.2); δ_C 167.26, 135.06, 131.16, 128.39, 127.06, 110.03, 68.17, 54.54, 32.97, 31.26, 19.07, 14.06; HRMS: 340.1563 (calcd. for $C_{18}H_{23}NO_4$ + Na 340.1525).

Isobutyl derivative **5c**. Mp 180–183 °C; υ_{max} 3338, 2956, 1656, 1132; δ_H 7.82–7.80 (2H, m), 7.51–7.41 (3H, m), 6.19–6.16 (1H, br d, *J* 10.0), 4.40–4.35 (4H, m), 2.60–2.56 (3H, d, *J* 13.2), 1.70–1.61 (5H, m), 1.49–1.41 (1H, m), 0.99–0.92 (2 × 3H, d, *J* 6.0); δ_C 167.19, 135.06, 131.16, 128.39, 127.08, 110.16, 68.17, 53.04, 38.03, 32.96, 24.54, 23.90, 21.66; HRMS: 354.1684 (calcd. for $C_{19}H_{25}NO_4$ + Na 354.1681).

Benzyl derivative **5d**. Mp 180–182 °C; υ_{max} 3329, 2960, 1656, 1315, 1132; δ_H 7.65–7.63 (2H, m), 7.47–7.10 (8H, m), 6.22–6.19 (1H, br d, *J* 9.9), 4.69–4.61 (1H, m), 4.43 (3H, br s), 3.33–3.26 (1H, dd, *J* 4.2, 14.4), 2.83–2.75 (1H, dd, *J* 10.2, 14.4), 2.63–2.58 (3H, d, *J* 12.9), 1.73–1.69 (3H, d, *J* 12.9); δ_C 167.06, 138.17, 135.03, 131.04, 129.41, 128.28, 128.03, 126.94, 126.01, 109.94, 68.24, 55.08, 34.98, 32.91; HRMS: 388.1583 (calcd. for $C_{22}H_{23}NO_4$ +Na 388.1525).

Allyl derivative **5e**. Mp 164–167 °C; υ_{max} 3330, 2959, 1656, 1132; δ_H 7.81–7.77 (2H, m), 7.51–7.39 (3H, m), 6.31–6.28 (1H, bd, *J* 9.9), 5.95–5.81 (1H, m), 5.10–4.98 (2H, m), 4.41–4.35 (4H, m), 2.68–2.55 (4H, m), 2.36–2.25 (1H, m), 1.72–1.68 (3H, d, *J* 12.9); δ_C 167.05, 134.95, 134.91, 131.12, 128.32, 126.97, 116.91, 109.72, 68.16, 53.95, 33.81, 32.83; HRMS: 338.1199 (calcd. for $C_{18}H_{21}NO_4$ + Na 338.1368).

Propargyl derivative **5f**. Mp 206–207 °C; υ_{max} 3328, 3238, 2958, 1641, 1132; δ_H 7.86–7.83 (2H, m), 7.53–7.41 (3H, m), 6.46–6.43 (1H, br d, *J* 9.9), 4.56–4.48 (1H, m), 4.42 (3H, br s), 2.81–2.52 (5H, m), 1.92 (1H, t, *J* 2.7), 1.72–1.68 (3H, d, *J* 12.9); δ_C 167.26, 134.83, 131.28, 128.39, 127.17, 109.32, 80.88, 69.64, 68.26, 52.87, 32.80, 19.35; HRMS: 336.1211 (calcd. for $C_{18}H_{19}NO_4$ + Na 336.1212).

Ethyl α-alkyl-α-(2,4,10-trioxaadamant-3-yl)acetates **7**

Methyl derivative **7a**. Oil; υ_{max} 2955, 1734, 1538, 1135; δ_H 4.42 (3H, br s), 4.23–4.18 (2H, q, *J* 7.2), 2.78–2.73 (1H, q, *J* 7.2), 2.61–2.58 (3H, d, *J* 13.2), 1.71–1.67 (3H, d, *J* 12.8), 1.29–1.22 (6H, m); δ_C 171.63, 109.78, 68.28, 60.44, 48.49, 32.74, 14.19, 11.01; HRMS: 265.1056 (calcd. for $C_{12}H_{18}O_5$ + Na 265.1052).

n-Propyl derivative **7b**. Oil; υ_{max} 2961, 1740, 1734, 1316, 1136; δ_H 4.42 (3H, br s), 4.29–4.14 (2H, m), 2.67–2.63 (1H, dd, *J* 3.6, 11.6), 2.61–2.58 (3H, d, *J* 12.8), 1.83–1.67 (5H, m), 1.32–1.18 (5H, m), 0.90 (3H, t, *J* 7.2); δ_C 171.09, 109.67, 68.24, 60.26, 54.64, 32.70, 28.11, 20.67, 14.21, 13.82; HRMS: 293.1364 (calcd. for $C_{14}H_{22}O_5$ + Na 293.1365).

Isobutyl derivative **7c**. Oil; υ_{max} 2958, 1741, 1630, 1133; δ_H 4.39 (3H, br s), 4.27–4.09 (2H, m), 2.74–2.70 (1H, dd, *J* 2.8, 12.0), 2.58–2.55 (3H, d, *J* 12.8), 1.82–1.77 (1H, m), 1.67–1.64 (3H, d, *J* 12.8), 1.51–1.41 (2H, m), 1.24 (3H, t, *J* 7.2), 0.88–0.84 (2 × 3H, d, *J* 4.8); δ_C 171.23, 109.88, 68.29, 60.33, 52.92, 34.80, 32.74, 26.09, 23.42, 21.39, 14.24. HRMS: 307.1528 (calcd. for $C_{15}H_{24}O_5$ + Na 307.1521).

Benzyl derivative **7d**. Mp 91–93 °C; υ_{max} 2959, 1737, 1316, 1133; δ_H 7.27–7.14 (5H, m), 4.46 (3H, br s), 4.13–3.98 (2H, m), 3.14–2.95 (3H, m), 2.65–2.62 (3H, d, *J* 12.8), 1.73–1.70 (3H, d, *J* 12.8), 1.04 (3H, t, *J* 7.2); δ_C 170.13, 139.11, 128.91, 128.17, 126.06, 109.50, 68.41, 60.34, 56.51, 32.75, 32.20, 14.03; HRMS: 341.1368 (calcd. for $C_{18}H_{22}O_5$ + Na 341.1365).

Allyl derivative **7e**. Oil; υ_{max} 2959, 1739, 1733, 1646, 1133; δ_H 5.79–5.69 (1H, m), 5.10–4.96 (2H, m), 4.43 (3H, br s), 4.25–4.16 (2H, m), 2.77–2.73 (1H, dd, *J* 5.2, 10.0), 2.61–2.51 (5H, m), 1.71–1.67 (3H, d, *J* 12.8), 1.25 (3H, t, *J* 7.2); δ_C 170.32, 135.33, 116.47, 109.47, 68.35, 60.47, 54.23, 32.71, 30.45, 14.28; HRMS: 291.1200 (calcd. for $C_{14}H_{20}O_5$ + Na 291.1208).

4-Methoxylbenzyl derivative **7f**. Oil; υ_{max} 2957, 1739, 1609, 1246, 1131; δ_H 7.11–7.09 (2H, d, *J* 8.8), 6.78–6.76 (2H, d, *J* 8.8), 4.45 (3H, br s), 4.14–4.00 (2H, m), 3.76 (3H, s), 3.07–2.90

(3H, m), 2.64–2.61 (3H, d, *J* 12.8), 1.72–1.69 (3H, d, *J* 12.8), 1.06 (3H, t, *J* 7.2); δ_C 170.29, 157.94, 131.12, 129.92, 113.63, 109.52, 68.44, 60.41, 56.82, 55.18, 32.79, 31.33, 14.13. HRMS: 371.1475 (calcd. for $C_{19}H_{24}O_6$ + Na 371.1471).

α-Alkyl-α-(2,4,10-trioxaadamant-3-yl)acetic acids **8**

Methyl derivative **8a**. Mp 107–109 °C; υ_{max} 2960, 1716, 1317, 1131; δ_H 4.47 (3H, br s), 2.78–2.72 (1H, q, *J* 7.2), 2.63–2.60 (3H, d, *J* 12.8), 1.76–1.73 (3H, d, *J* 12.8), 1.26–1.24 (3H, d, *J* 7.2); δ_C 172.24, 110.05, 68.69, 48.47, 32.53, 10.48; HRMS: 237.0738 (calcd. for $C_{10}H_{14}O_5$ + Na 237.0739).

n-Propyl derivative **8b**. Mp 97–99 °C; υ_{max} 2962, 1710, 1315, 1132; δ_H 4.47 (3H, br s), 2.71–2.67 (1H, dd, *J* 4.4, 10.0), 2.65–2.62 (3H, d, *J* 14.2), 1.81–1.66 (5H, m), 1.49–1.24 (2H, m), 0.92 (3H, t, *J* 7.2); δ_C 173.18, 109.76, 68.59, 54.55, 32.59, 27.89, 20.65, 13.85; HRMS: 243.1384 (calcd. for $C_{12}H_{18}O_5$ + H 243.1232).

Isobutyl derivative **8c**. Mp 137–139 °C; υ_{max} 2958, 1709, 1315, 1132; δ_H 4.47 (3H, br s), 2.79–2.76 (1H, dd, *J* 2.8, 10.8), 2.65–2.61 (3H, d, *J* 12.4), 1.76–1.52 (6H, m), 0.93–0.90 (2 × 3H, d, *J* 4.8); δ_C 173.14, 109.88, 68.59, 52.80, 34.63, 32.60, 25.82, 23.37, 21.29; HRMS: 279.1202 (calcd. for $C_{13}H_{20}O_5$ + Na 279.1208).

Benzyl derivative **8d**. Mp 96–98 °C; υ_{max} 2960, 1715, 1316, 1130; δ_H 7.25–7.16 (5H, m), 4.46 (3H, br s), 3.14–2.94 (3H, m), 2.64–2.61 (3H, d, *J* 12.8), 1.74–1.71 (3H, d, *J* 12.8); δ_C 173.05, 138.87, 128.94, 128.29, 126.24, 109.54, 68.66, 56.44, 32.61, 31.92; HRMS: 313.1031 (calcd. for $C_{16}H_{18}O_5$ + Na 313.1052).

Allyl derivative **8e**. Mp 91–93 °C; υ_{max} 2962, 1718, 1318, 1132; δ_H 5.86–5.76 (1H, m), 5.13–5.01 (2H, m), 4.48 (3H, br s), 2.79–2.76 (1H, dd, *J* 4.4, 10.4), 2.65–2.45 (5H, m), 1.76–1.73 (3H, d, *J* 13.2); δ_C 172.88, 134.98, 116.89, 109.53, 68.64, 54.36, 32.58, 30.23; HRMS: 241.0908 (calcd. for $C_{12}H_{16}O_5$ + H 241.1076).

4-Methoxybenzyl derivative **8f**. Mp 123–125 °C; υ_{max} 2958, 1733, 1246, 1131; δ_H 7.14–7.12 (2H, d, *J* 8.4), 6.79–6.77 (2H, d, *J* 8.4), 4.48 (3H, br s), 3.76 (3H, s), 3.10–2.91 (3H, m), 2.66–2.63 (3H, d, *J* 12.8), 1.76–1.72 (3H, d, *J* 12.8); δ_C 172.62, 157.82, 130.62, 129.72, 113.52, 109.34, 68.65, 56.53, 54.91, 32.42, 30.85; HRMS: 343.1176 (calcd. for $C_{17}H_{20}O_5$ + Na 343.1158).

α-Alkyl-α-(2,4,10-trioxaadamant-3-yl)acetamides **9**

Methyl derivative **9a**. Mp 175–176 °C; υ_{max} 3441, 2947, 1667, 1133; δ_H 7.29 & 6.57 (2 × 1H, s), 4.44 (3H, br s), 2.65–2.59 (4H, m), 1.75–1.72 (3H, d, *J* 12.8), 1.26–1.24 (3H, d, *J* 7.2); δ_C 174.47, 110.01, 68.28, 49.83, 32.73, 11.57; HRMS: 236.0894 (calcd. for $C_{10}H_{15}NO_4$ + Na 236.0899).

n-Propyl derivative **9b**. Mp 156–158 °C; υ_{max} 3437, 2961, 1663, 1130; δ_H 6.36 & 5.69 (2x1H, s), 4.43 (3H, br s), 2.62–2.58 (3H, d, *J* 12.8), 2.52–2.48 (1H, dd, *J* 2.4, 11.2), 1.80–1.57 (5H, m), 1.47–1.26 (2H, m), 0.92 (3H, t, *J* 7.2); δ_C 173.97, 109.85, 68.28, 56.32, 32.74, 28.99, 20.61, 14.01; HRMS: 264.1223 (calcd. for $C_{12}H_{19}NO_4$ + Na 264.1212).

Isobutyl derivative **9c**. Mp 179–181 °C; υ_{max} 3429, 2957, 2929, 1665, 1128; δ_H 6.26 & 5.28 (2 × 1H, s), 4.41 (3H, br s), 2.60–2.56 (4H, m), 1.71–1.68 (3H, d, 12.8), 1.66–1.48 (3H, m), 0.91–0.88 (2x3H, d, *J* 6.0); δ_C 173.95, 109.96, 68.28, 54.49, 35.76, 32.76, 25.64, 23.65, 21.36; HRMS: 278.1359 (calcd. for $C_{13}H_{21}NO_4$ + Na 278.1368).

Benzyl derivative **9d**. Mp 146–148 °C; υ_{max} 3467, 2928, 1664, 1130; δ_H 7.21–7.11 (5H, m), 6.14 & 5.45 (2 × 1H, br s), 4.44 (3H, br s), 3.15–3.12 (1H, dd, *J* 4.0, 14.0), 2.78–2.74 (1H, dd, *J* 4.0, 10.8), 2.62–2.59 (3H, d, *J* 12.8), 1.73–1.70 (3H, d, *J* 12.8); δ_C 172.49, 139.48, 128.99, 128.18, 126.04, 109.68, 68.42, 57.70, 32.75, 32.51; HRMS: 312.1219 (calcd. for $C_{16}H_{19}NO_4$ + Na 312.1212).

Allyl derivative **9e**. Mp 132–134 °C; υ_{max} 3448, 2960, 1667, 1127; δ_H 6.34 & 5.59 (2 × 1H, s), 5.89–5.78 (1H, m), 5.12–4.99 (2H, m), 4.44 (3H, br s), 2.62–2.53 (5H, m), 2.46–2.38 (1H, m), 1.75–1.71 (3H, d, *J* 13.2); δ_C 172.82, 135.62, 116.30, 109.62, 68.35, 55.95, 32.72, 31.15; HRMS: 262.1058 (calcd. for $C_{12}H_{17}NO_4$ + Na 262.1055).

4-Methoxybenzyl derivative **9f**. Mp 195–197 °C; υ_{max} 3468, 2957, 1682, 1131; δ_H 7.17–7.15 (2H, d, *J* 8.4), 6.79–6.77 (2H, d, *J* 8.4), 6.16 & 5.43 (2 × 1H, s), 4.45 (3H, br s), 3.75 (3H, s), 3.11–3.07 (1H, dd, *J* 3.6, 14.0), 2.96–2.90 (1H, dd, *J* 11.2, 14.0), 2.75–2.71 (1H, dd, *J*=3.6, 11.2), 2.63–2.60 (3H, d, *J* 12.8), 1.75–1.71 (3H, d, *J* 12.8); δ_C 172.59, 157.87, 131.43, 129.92, 113.62, 109.66, 68.40, 58.00, 55.10, 32.76, 31.68; HRMS: 342.1316 (calcd. for $C_{17}H_{21}NO_5$ + Na 342.1317).

N-Methoxycarbonyl-1-alkyl-1-(2,4,10-trioxaadamant-3-yl)methylamines **10** (Hofmann reaction products)

Methyl derivative **10a**. Mp 130–132 °C; υ_{max} 3375, 2960, 1722, 1716, 1527, 1134; δ_H 5.01–4.99 (1H, d, *J* 7.6), 4.40 (3H, br s), 3.84–3.78 (1H, m), 3.66 (3H, s), 2.59–2.56 (3H, d, *J* 12.8), 1.72–1.69 (3H, d, *J* 12.8), 1.16–1.15 (3H, d, *J* 6.8); δ_C 156.65, 109.72, 68.11, 52.42, 51.90, 32.84, 14.95; HRMS: 266.1042 (calcd. for $C_{11}H_{17}NO_5$ + Na 266.1004).

n-Propyl derivative **10b**. Mp 137–139 °C; υ_{max} 3394, 2962, 1722, 1525, 1241; δ_H 4.82–4.79 & 4.68–4.65 (0.8 & 0.2H, d, *J* 10.4, 9.6), 4.39 (3H, br s), 3.74–3.67 (4H, m), 2.59–2.56 (3H, d, *J* 12.8), 1.77–1.67 (4H, m), 1.46–1.24 (3H, m), 0.90 (3H, t, *J* 7.2); δ_C 157.34, 109.88, 68.07, 56.28, 51.98, 32.87, 31.10, 18.91, 13.90; HRMS: 294.1320 (calcd. for $C_{13}H_{21}NO_5$ + Na 294.1317).

Isobutyl derivative **10c**. Mp 116–118 °C; υ_{max} 3390, 2956, 1728, 1133; δ_H 4.73–4.71 & 4.60–4.58 (0.8 & 0.2H, d, *J* 10.4, 9.6), 4.36 (3H, br s), 3.80–3.65 (4H, m), 2.56–2.53 (3H, d, *J* 12.8), 1.67–1.58 (4H, m), 1.52–1.46 (1H, m), 1.30–1.23 (1H, m), 0.91–0.87 (2x3H, d, *J* 6.8);

δ_C 157.30, 110.00, 68.07, 54.74, 51.99, 37.86, 32.88, 24.25, 23.82, 21.39; HRMS: 308.1474 (calcd. for $C_{14}H_{23}NO_5$ + Na 308.1474).

Benzyl derivative **10d**. Mp 126–128 °C; υ_{max} 3390, 2958, 1731, 1521, 1132; δ_H 7.21–7.09 (5H, m), 4.79–4.77 (1H, d, *J* 10.0), 4.37 (3H, br s), 4.04–3.99 & 3.83–3.76 (0.8 & 0.2H, m), 3.48 & 3.23 (2.4 & 0.6H, s), 3.15–3.12(1H, m), 2.57–2.53 (4H, m), 1.67–1.64 (3H, d, *J* 12.8); δ_C 156.93, 138.31, 129.33, 128.02, 125.97, 109.82, 68.21, 57.11, 51.96, 35.06, 32.88; HRMS: 342.1315 (calcd. for $C_{17}H_{21}NO_5$ + Na 342.1317).

Allyl derivative **10e**. Mp 118–119 °C; υ_{max} 3398, 2958, 1722, 1717, 1132; δ_H 5.87–5.77 (1H, m), 5.08–5.00 (2H, m), 4.86–4.84 (1H, d, *J* 10.0), 4.40 (3H, br s), 3.85–3.79 (1H, m), 3.67 (3H, s), 2.60–2.52 (4H, m), 2.19–2.11 (1H, m), 1.71–1.68 (3H, d, *J* 13.2); δ_C 157.09, 134.91, 116.92, 109.65, 68.14, 55.85, 52.06, 33.79, 32.85; HRMS: 292.1157 (calcd. for $C_{13}H_{19}NO_5$ + Na 292.1161).

4-Methoxybenzyl derivative **10f**. Oil; υ_{max} 3376, 2958, 1731, 1515, 1131; δ_H 7.13–7.11 (2H, d, *J* 8.4), 6.80–6.78 (2H, d, *J* 8.4), 4.84–4.82 (1H, d, *J* 10.4), 4.42 (3H, br s), 4.03–3.99 (1H, m), 3.55 & 3.33 (2.4 & 0.6H, s), 3.13–3.10 (1H, m), 2.61–2.50 (4H, m), 1.72–1.68 (3H, d, *J* 12.8); δ_C 157.83, 156.96, 130.33, 130.23, 113.45, 109.82, 68.19, 57.29, 55.08, 51.94, 34.11, 32.88; HRMS: 372.1405 (calcd. for $C_{18}H_{23}NO_6$ + Na 372.1423).
